# Importance of performing SPECT/CT in thyroid Cancer patients with central neck uptake in whole body Iodine scan: A case report

**DOI:** 10.22038/aojnmb.2026.90936.1663

**Published:** 2026

**Authors:** Nasrin Raeisi, Mohammad Ahmadi, Haniye Elahifard, Amin Saber Tanha

**Affiliations:** Nuclear Medicine Research Center, Mashhad University of Medical Sciences, Mashhad, Iran

**Keywords:** Whole body iodine scan SPECT/CT, Thyroid cancer, Pitfalls, Differentiated thyroid carcinoma

## Abstract

The planar whole body iodine scan (WBIS) has traditionally been used as the primary imaging technique for assessment of patients with thyroid cancer. This case report examines the imaging findings of a 61-year-old male with follicular thyroid carcinoma who underwent total thyroidectomy and received adjuvant radioactive iodine (RAI) treatment. A planar post-ablation WBIS revealed the commonly seen pattern of multiple foci of iodine uptake in the central neck region suggesting postsurgical thyroid remnant, thyroglossal duct remnant and central lymph node metastasis. However, single photon emission computed tomography (SPECT) / computed tomography (CT) imaging excluded lymph node metastasis and revealed iodine contamination over the mentum superimposed on the thyroid bed. This case report highlights the importance of utilizing SPECT /CT even for patients with apparently central neck uptake to prevent incorrect staging and treatment planning. In this report, we also present a review of the literature highlighting various pitfalls that can affect the interpretation of the WBIS and potentially result in false-positive findings in the neck region.

## Introduction

 Thyroid carcinoma is the most common endocrine malignancy and its incidence has been remarkably increased in the recent decades (1). The primary treatment for patients with differentiated thyroid carcinoma (DTC) is thyroidectomy followed by radioactive iodine (RAI) therapy in high risk patients (2). A post-ablation whole body iodine scan (WBIS) is mandatory for all patients undergoing RAI ablation and not only reveals local or distant metastasis, but also gives more information about the iodine avidity of the disease (3). The detection of lymph node or distant metastasis is the main tool for risk stratification. The extent and severity of the treatment highly depends on the risk of disease which is usually defined by TNM staging. The WBIS plays the main role for detection of local or distant metastasis, which is traditionally performed as planar images. However, planar imaging has a number of limitations and single photon emission computed tomography (SPECT)/ computed tomography (CT) could potentially change TNM staging in a number of patients which could have therapeutic implications (4-6). In this case report we present a patient with suspicion of lymph node metastasis on planar scan which is excluded by SPECT/CT imaging and changed N category of TNM staging.

## Case presentation

 A 61-year-old man with history of neck swelling for 4 months, underwent fine-needle aspiration (FNA) of a thyroid nodule in the right lobe that turned to be papillary thyroid carcinoma (PTC). Total thyroidectomy and central lymph node dissection was performed. The pathology examination showed a 3.5×2.4×2.2 cm follicular carcinoma in the right lobe with a few foci of poorly differentiated thyroid carcinoma. T and N category was pT2 and pN0a (none of the four resected central neck lymph nodes showed involvement). He was referred to our department for I-131 therapy. Upon presentation, the biochemical profile was as follows: serum thyroglobulin (S.TG): 2.59 ng/mL, serum anti-thyroglobulin (S. anti-TG): 20 IU/L, and thyroid-stimulating hormone (TSH) >78.5 mIU/L. Due to high risk features, he was admitted and 5.55 GBq of I-131 was administered as adjuvant therapy. A post-ablation planar WBIS was conducted seven days later utilizing a dual-head gamma camera (GE; Discovery NM/CT 670), equipped with high-energy collimators and configured with a 20% energy window set at 364 kiloelectron volts (keV). The scan was acquired in continuous acquisition mode at a table speed of 8 cm/min, employing automated body contour detection and recorded in a matrix size of 1024×256 (Figure 1-A). The planar scan revealed multiple foci of radio-iodine uptake in the central neck region corresponding to the thyroglossal duct remnant (Figure 1-A, blue arrow), thyroid bed remnant (Figure 1-A, green arrow), and a focus inferior to thyroid bed, suggestive of lymph node metastasis (Figure 1-A, red arrow). SPECT imaging was performed from the neck region in a step-and-shoot mode (30 seconds/stop), 64 frames/head , using a noncircular orbit, over 360° (180º per head) saved in a 64×64 matrix and were reconstructed using iterative reconstruction (IR) method. CT acquisition with a tube voltage of 120 kV, tube current of 140 mAs and reconstructed slice width of 2.5 mm was performed subsequent to SPECT for attenuation correction, and anatomical localization. Contrary to findings of planar image, SPECT/CT images revealed that the uptake initially thought to be in the thyroid bed was actually an iodine contamination over the mentum. Furthermore, the focus previously misinterpreted as lymph node metastasis in planar WBIS, was confirmed to be the thyroid remnant on the left thyroid bed (Figure 1-B). So the patient was down staged from stage II (T2N1M0) to stage I (T2N0M0).

**Figure:**
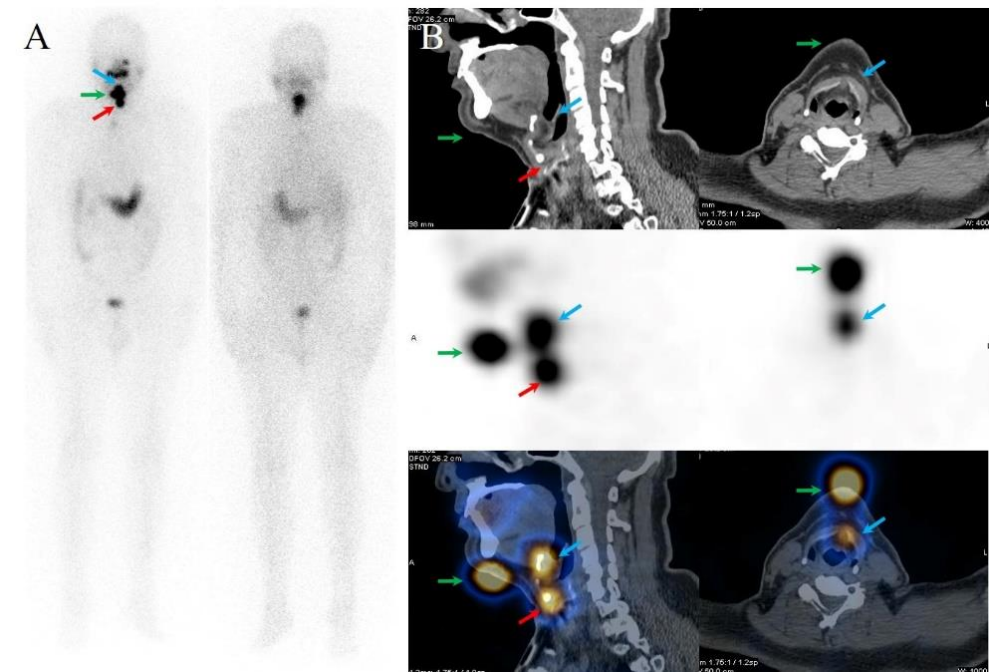


## Discussion

 Differentiated thyroid cancer, considered as the most prevalent endocrine malignancy, has increasingly become a significant public health concern. Globally, it ranked 7th most common cancer overall and 5th most common cancer in female, with projections indicating a potential increase of approximately 30 percent between 2019 and 2030 (7). Surgery remains the primary modality of treatment in these patients; however, internal radiation therapy utilizing RAI in high risk patients presents as important therapeutic necessity and the post-ablation WBIS is one of the main tools for risk stratification (8-12).

 Traditionally WBIS is performed as planar imaging, however, false-positive findings and pitfalls are common and may affect risk stratification and treatment planning (13–18). Most of the patients with DTC have a small post-surgical thyroid remnant that can be seen as central neck uptake in a planar whole body iodine scan. Commonly, central neck activity is considered as thyroid remnant and/or thyroglossal duct remnant, and any extension beyond thyroid bed may suggest a lymph node metastasis (19). Additional views such as lateral and/or oblique images may be helpful in occasional patients, however, due to poor spatial resolution of high-energy imaging, it is not easy to localize the lesion correctly in all of the patients. Although nuclear physicians are more prone to interpret central neck uptake as a thyroid bed remnant, false positive findings in this region might be seen and might result in incorrect diagnosis and treatment of the patient.

 Numerous false positive iodine uptake has been reported in many organs either due to physiologic uptake or pathologic conditions. We summarized false positive uptake in the neck region in Table 1 (20-43). Our case report showed that saliva contamination may superimpose on the thyroid bed and be misinterpreted as a remnant or lymph node metastasis. Considering extensive cases of false positive in the neck region, SPECT/CT imaging could be invaluable for better delineation of any uptake in this region. This approach aligns with the recommendations in the latest edition of the American Thyroid Association (ATA) guidelines, which indicate that SPECT/CT may be performed when available in conjunction with diagnostic or post-treatment WBIS (44).

**Table 1:** Neck Iodine Activity (Review of the Literature)

	**Physiologic**			**Pathologic**		
**Thyroid-related uptakes**	**Ectopic thyroid**			Tumoral remnant in thyroid bed		
	Pyramidal lobe (21,22)			Lymph node metastasis		
	Aberrant tissue (21,22)			Cervical vertebrae metastasis(24)		
	Lingual thyroid (21,22)			Cutaneous metastasis (25)		
	Esophageal thyroid (23)					
	Intra-tracheal thyroid (23)					
	**Post-surgical thyroid remnant** (21)					
**Non-thyroidal uptakes**	**Physiologic**					
	Salivary glands (21,26–28)					
	Nasopharynx (26,28)					
	Esophageal retention of iodine containing secretions (21,29)					
	**Pathologic**					
	**Infection/** **Inflammation**	**Benign Tumors**	**Malignant tumors**	**Trauma**	**Cystic degeneration**	**Other**
		Warthin’s tumor (22,28,31)	Salivary gland adenocarcinoma (21,33)	Post traumatic superficial scab/wound (foreign body)(31,33)	Lymphoepithelial cyst of parotid gland (Sub-parotidian lymphoepithelial cyst) (22,33)	Barret’s esophagous (28)
	Sialadenitis (22,30)	Salivary gland oncocytoma (27,31,34)	metastasis from other carcinoma (31)	Biopsy acupuncture sites (23,26,31)	Thyroglossal duct cyst (31,35)	Fistula tract (41)
	Dental disease (22,23,26,28)	Pleomorphic adenoma (35)	Squamous cell carcinoma (31)	Surgical suture(31)	Bone cysts (40)	Suture granuloma(41)
	Oral disease (22)	Spinal cord meningioma (33)		Surgical clips (28)		Laryngocele (21,42)
	Folliculitis (22,26,31)	Cavernous angioma (27,31)		Site of tracheotomy (31)		Sialolithiasis (27)
	TM joint effusion(32)	Vertebral hemangioma (31,36)		Post-operative seroma (38)		Salivary gland duct ectasia(27)
	Degenerative/inflammatory changes in Cervical vertebrae Skin burn(23,26,33)	Osteoid osteoma (31)		Bone fracture (39)		Dental amalgam (27)
	Sebaceous cysts (23,29)	Lipoma (36)				Poorly dissolved ^131^I capsule (21,23)
	Psoriatic plaque (23)	Nurilemoma (26)				Tracheostomy tube (26,28)
		Dermoid cyst (37)				Esophageal stricture (21,23,28)
						Zenker’s diverticulum (21,23,28,31)
						Carotid ectasia (21–23)
External contamination*	**Body secretions**					
	Saliva (22,23,26,43)					
	Nasal secretion (22,23,26)					
	Sweat (20,22,26,28,43)					
	Tracheobronchial secretions (22,26)					
	Urine (23,26,28)					
	Breast milk (23,26,28)					
	Fecal (26)					
	Vomitus (23,26)					

 In the present case, iodine contamination over the mentum, was superimposed on the thyroid bed and led to the misdiagnosis of lymph node metastasis in planar images. It was considered a TNM stage II disease, while SPECT/CT excluded lymph node metastasis and downstaged it into TNM stage I. This case report underlines the importance of acquiring SPECT/CT images and using anatomic landmarks even in the common cases of central neck uptake in WBIS.

## Conclusion

 This case highlights the essential role of SPECT/CT in distinguishing true central neck uptake from common pitfalls. Accurate localization in WBIS supports better risk stratification and treatment planning. We recommend SPECT/CT for all DTC patients if available, with careful consideration of potential false positives in the neck region.
